# Atmospheric Pollution Mapping of the Yangtze River Basin: An AQI-Based Weighted Co-Word Analysis

**DOI:** 10.3390/ijerph17030817

**Published:** 2020-01-28

**Authors:** Yu Song, Bingrui Liu, Xiaohong Chen, Jia Liu

**Affiliations:** 1 School of Public Administration, Huazhong Agriculture University, Wuhan 430072, China; songyu@mail.hzau.edu.cn; 2 School of Information and Safety Engineering, Zhongnan University of Economics and Law, Wuhan 430073, China; audrey514@126.com; 3 Institute of Big Data and Internet Innovation, Hunan University of Commerce, Changsha 410205, China; cxhcsu@126.com

**Keywords:** air pollution incidents, co-word analysis, Yangtze River Basin, clustering

## Abstract

The purpose of this paper is to analyze the characteristics and human effects of atmospheric pollution in the Yangtze River Basin (YRB). An AQI(Air Quality Index)-based weighted co-word method is applied to explore the characteristics of keywords taken from the data, using authoritative media sources and government reports. Hierarchical clustering techniques are utilized to classify and visualize the keywords and display the different types of incidents. The results reveal the following four main clusters: enterprise pollution, coal-burning pollution, traffic pollution, and air pollutants. Cluster 1 is divided into 7 sub-clusters to offer powerful insight into the structural characteristics of industrial activities. This study is one of the first attempts to use a bibliometric approach to visualize the underlying and interconnected sub-clusters from grey data. It also provides an atmospheric pollution mapping for formulating government policies by understanding the human effects of air pollution incidents.

## 1. Introduction

Addressing air pollution while advancing economic development tends to be a conundrum for developing countries [1,2]. Air pollution is also a disquieting phenomenon affecting the environment as well as presenting climate and human health problems to communities [3]. It is influenced by both natural and anthropogenic factors, while human activities are the primary causes [4]. Recognizing the main causes and characteristics of atmospheric issues is the key to effectively easing the perceived tension.

China, the largest developing country in the world, has experienced rapid economic development in the past decades. Metropolitan areas along the Yangtze River, especially in the Yangtze River Basin (YRB), account for more than 40% of GDP and 30% of the population in China [5]. A national outlook for achieving sustainable development is implemented by the State Council to emphasize the environmental issues in the YRB. However, this region has also suffered from severe pollution due to human activities [6]. Pollutants emitted into the atmosphere are mainly from the increasing consumption of fossil fuels (e.g., NO_x_, SO_2_, CO, CH_x_) [7,8]. Excessive concentration of the population, low energy efficiency, and increasing gas emissions also cause complex air pollution incidents in the whole region [9,10]. Specifically, the cities in the lower-middle reaches of the YRB are always covered by fog and haze, indicating a novel and gradually concerning type of air pollution. The deteriorating situation not only poses a great threat to the local residents but also affects sustainable development [11,12].

A set of unsettled issues need to be urgently answered. For instance, what are the main types of spatial patterns of air pollution in the YRB? What characteristics and human effects degrade the local atmosphere? What are the significant contributors to environmental deterioration and the interaction effect? Analyzing the solutions of the aforementioned questions and the main causes of air pollution to promote the sustainable development of the YRB have been hotly debated by experts, scholars, and policymakers in China [13,14]. A number of researchers suggest that meteorological factors promote the deterioration and evolution of air pollution [15], especially rainfall, wind, and barometric pressure [16,17,18], etc. In addition, some topographical conditions are discussed to explain the diffusion and clustering of conventional pollutants (e.g., NOx, SO_2_, CO, PM_10_) [19]. Regarding human activities, current studies state that population concentration, industrial activities, fossil fuel consumption, agricultural production, and motor vehicle exhaust fumes are the main sources of atmospheric pollution [20,21]. To understand the formation and transport of air pollutants from urban to national areas, emission inventories are discussed for modeling the formulation of air pollution [22]. Meanwhile, some factors such as urban conglomerations, economic and social development, industrial structure, land planning, etc., can also lead to deteriorating atmospheric conditions [23,24]. Some other studies also focus on individual pollutants or certain sectors [25]. The existing studies pay more attention to explicating what contributes to atmospheric pollution of the YRB considering meteorological and human factors [26]. 

Statistical analysis methods are frequently applied to describe the pollution sources, especially though time series analysis and spatial analysis [27,28]. Mathematical research has revealed single factors, such as spatiotemporal variation, meteorological factors, pollution sources, socioeconomic factors, etc. [29] Regarding the environmental incidents, researchers focus more on environmental quality evaluation systems in the metropolis of the YRB [30,31]. The analysis of deep causes (not only from objective data) and the interaction of factors are critical, but often-overlooked, components of air pollution, which may contribute to biased conclusions. Researchers pay more attention to existing environmental monitoring data [32,33]. However, it seems less common to thoroughly explore the law of air pollution incidents. 

Bibliometrics is defined as “the quantitative research of published units, or of bibliographic units, or of the surrogates for either” [34]. It can be extended to any quantitative measures or analyses applied to air pollution incidents from government reports, news, books, journal articles, etc. Co-word analysis is a content technique that is effective in structuring the relationship between keyword items in textual data [13]. The visualization of the results through keywords based on the co-word method can extract and cluster the characteristics and human effects of air pollution incidents [35]. Meanwhile, the keywords are recognized from the perspective of big data to obtain the main factors, and targeted suggestions for regional development are proposed.

Hence, the authors attempt to solve the questions mentioned above in this article. Our work is organized as follows. The first step is to introduce a novel method, an AQI(Air Quality Index)-based weighted co-word method, to analyze the characteristics and human effects of air pollution incidents. An overview of the database crawled from the Web in recent years is implemented. The “Results and Discussion” section presents a statistical analysis of air pollution incidents in the YRB. Moreover, taking AQI as the weight, high-frequency keywords are clustered and visualized in multidimensional space to discuss the intrinsic link between different incidents. The “Conclusion” section concludes with suggestions for the future work of environmental protection in this region. 

## 2. Methodology

Co-word analysis is an effective method in structuring relationships and mapping the strength between information items in a textual unit [35]. It is widely used in bibliometrics, which provides quantitative insight into a large amount of structured text [36,37]. It always provides insight into the growth of literature and the trend of knowledge within a specified field by analyzing key information extracted from the database, such as citations, authors, keywords, etc. [38,39] Meanwhile, this methodology is innovatively used in analyzing the unstructured texts. Wu (2018) utilized a co-word analysis of the keywords extracted from the Web, using texts of environmental loss-related chemical accidents, and used Pearson’s correlation coefficient to examine the internal attributes [40]. Through the bibliometric mapping, each research unit (book, paper, etc.) is assumed to be characterized by a set of important keywords [41]. “Bibliometric”, based on co-word analyses, can be treated as a mining tool to visualize different relationships, significance, and centrality, thus elaborating on the underlying causes [37].

**Figure 1:**
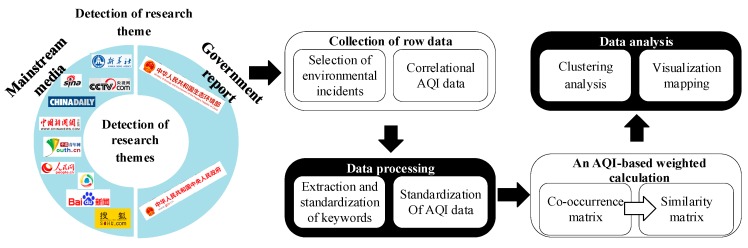
The process of the AQI-based weighted co-word analysis.

A number of researchers utilize diversified approaches such as descriptive analysis [15] and systems dynamics [11,42] to present actual data, especially from sensors and other types of measurements. However, the intrinsic relevance of characteristics that contribute to air pollution is rarely considered. The goal of the co-word method in this research is to interconnect the significant characteristics or causes among environmental incidents by visualizing the “distance between keywords”. Few current studies utilize this correlation perspective to the area of accident analysis but always report either the temporal variation or spatial variation of air pollution. The inspiration is from bibliometrics. Texts on web pages such as mainstream media news and government reports related to atmospheric pollution in the YRB are considered as analysis units. The keywords are extracted from the text reflecting the key characteristics or causes of a single environmental incident. Those keywords can be calculated to analyze the correlation between different incidents and dig into the internal mechanism. Our approach draws on the study of [37] by focusing along the following lines (in Figure 1).

### 2.1. Detection of the Research Theme

The aim of this paper is to map the effects of atmospheric pollution and human factors in the YRB. The authors mainly concentrate on the collection and analysis of air pollution incidents. Reliable news from mainstream media provides detailed information and the latest statistics. Meanwhile, the environmental-related accident reports may include overall data or statements of atmospheric effects. Thus, these two kinds of grey data are treated as the research sample to extract the characteristics of atmospheric pollution incidents. The important news websites and official websites are selected as the target databases due to their authority and integrity, such as Baidu News, Sina News, Google News, Xinhua News, the website of the Ministry of Ecology and Environment, and the State Council. 

The headlines and texts of the news are searched with the theme keywords such as “the Yangtze River”, “air pollution”, “particle emissions”, “waste gas emissions” and “enterprise emissions”. As China has witnessed long-term smog weather in the YRB and the relevant reports have increased gradually from late 2014, the dynamic changes of AQI and various pollutants are recorded from then on. Thus, the time range of the sample search is defined from September 1, 2014 to August 30, 2019, correspondingly. The incidents mainly concentrate on 11 provinces in the YRB to achieve the spatial variation of pollution samples, including Sichuan, Yunnan, Chongqing, and Guizhou (the upper reaches); Hunan, Hubei, and Jiangxi (the middle reaches); Jiangsu, Anhui, Zhejiang, and Shanghai (the lower reaches). 

### 2.2. Collection of Raw Data

A total of 3677 items are finally obtained as initial samples. Texts of two distinct emotions, positive (related to environmental policies on the official websites) and negative (related to pollution incidents), are organized. Screening out negative ones manually and eliminating the irrelevant data, typical incidents are then selected. The AQI data of each incident are observed as keyword weight, which is taken from the China Air Quality Online Monitoring and Analysis Platform. In order to eliminate the influence of meteorological complexity, the data observation points are compared with the location of incidents. The recording time of AQI is also based on the time that the incident occurs, approximately implying the causation between the sample and the effect of air condition. Then, by deleting and merging the redundant data, a new database of 385 air pollution incidents with accurate AQI data is formed.

### 2.3. Data Processing

Meanwhile, keyword extraction is implemented through text mining. Keywords such as center words and high-frequency words are extracted. This process is also conducted manually by authors and experts in a non-parametric manner. Then, keywords of the same meaning or concept are standardized. Words with lower frequencies and unclear expressions are removed, such as unrelated verbs. After modification, a total of 63 keywords of which the frequency is more than 3 are reserved as main characteristics. However, in order to reflect some specific types of reported enterprises with relatively serious pollution, the keywords with low-frequency keywords are selected such as “building materials enterprises”, “buffing process”, etc.

### 2.4. An AQI-Based Weighted Calculation

The traditional calculation of the similarities between keywords is according to the co-occurrence matrix. Only a simple mathematical addition is calculated through cross-identification of data in the matrix. Due to the complex nature of common atmospheric pollution, researchers have difficulty addressing traits associated with various incidents. Few studies focus on the correlation analysis to compare the connections between contents in the grey text (e.g., social media messages, government reports) [43,44]. 

After the data processing, an AQI-based weighted calculation of the occurrence and similarity is conducted to deduce the characteristics and human effects of atmospheric pollution. The transformation of the keywords list to the AQI-based correlation matrix can be achieved through two steps: 

1) By counting the co-occurrence frequency of the high-frequency keywords, a basic co-word matrix can be formed. To differentiate the impact of each sample, the AQI data *α_i_* of incident *i* is standardized and treated as the weight coefficient of each incident [45]. Then, the AQI-based occurrence frequency of a certain keyword can be also equal to $\alpha_{i}^{AQI}$, not equal to 1.

(1) $$\alpha_{i}^{AQI}=\alpha_{i}/\sum_{i=1}^{N}\alpha_{i}$$

where $\alpha_{i}^{AQI}$ denotes the standardized weighted coefficient; $\alpha_{i}$ is the monitoring data of AQI after the incident occurs; N is the number of air pollution incidents.

2) Based on the modified occurrence frequencies of keywords, the basic co-occurrence matrix can be transformed into the AQI-based co-occurrence matrix.

(2) $$O_{ab}^{AQI}=\sum_{i=1}^{N}\alpha_{i}*o_{ab}^{i}$$

where $O_{ab}^{AQI}$ denotes the AQI-based co-occurrence of the keyword *a* and *b*. $o_{ab}^{i}=1$ means the keyword a and b appear in the sample i; otherwise, $o_{ab}^{i}=0$.

Pearson’s correlation is employed to eliminate the data deviation caused by different occurrence characteristics. Based on the AQI-based co-occurrence matrix, The AQI-based Pearson’s correlation matrix could be calculated. 

### 2.5. Clustering and Visualization

Hierarchical Clustering (HC) is a traditional method of dimension reduction [46]. This paper utilizes HC to classify the 63 high-frequency keywords and genealogy is formed by the software SPSS. Utilizing the vertical line to cut the keyword tree, the number of intersection points indicates the number of groups. Clustering the keywords and extracting common features between different groups tends to generalize the commonality of air pollution incidents in the YRB. The “vertical line” can be moved smoothly to reclassify the whole map and distinguish different categories of features. 

Then, in order to elaborate the correlation of different clusters based on HC, network analysis is utilized based on software Ucinet to visualize the keyword group in a data map [46]. The position of keywords is checked using the modified Pearson’s correlation coefficient to obtain general characteristics. The near/far distance between two keywords indicates the higher/lower similarity of them. Meanwhile, the high centrality indicates multiple lines of links to the core keywords. Then, all keywords are clustered in the multidimensional space using the MDS (Multi-dimensional Scaling) method to display different types in a visualization network. 

## 3. Results and Discussion

### 3.1. Descriptive Statistics

**Figure 2:**
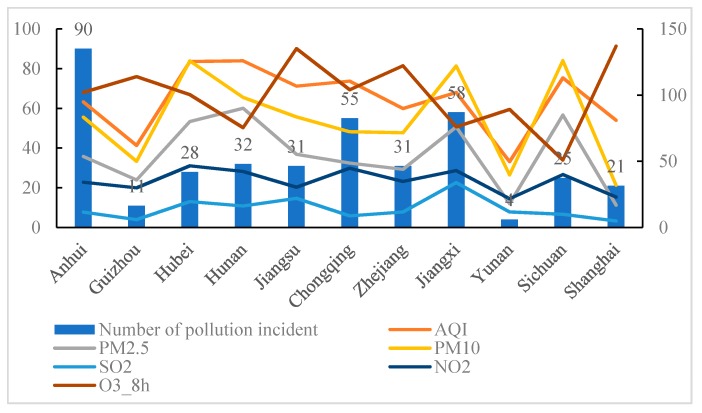
Number of pollution incidents and monitoring data in the Yangtze River Basin (YRB).

The negative effects of air pollution have long been recognized [47]. However, the relationship between rapid urbanization and environmental protection is emphasized to be a difficult issue. According to 386 air pollution incidents collected, the environmental problems in the YRB are very serious. Figure 2 presents the relationship between the occurrence of incidents and pollutants in 11 provinces and cities from 2014 to 2019. 

To some extent, the high speed of development in economies and industries is not completely related to serious pollution in the YRB [15]. The cities with the fastest economic development are mainly located in the middle and lower reaches. However, it should be noted that three provinces, namely Anhui, Jiangxi, Chongqing (covering the whole basin), are comparatively higher than the rest areas. The monitoring data (AQI, PM_2.5_, PM_10_, SO_2,_ NO_2_, and O_3_) show a strong correlation between the occurrence frequency of incidents, especially in Hubei, Jiangxi, and Sichuan. However, some underdeveloped regions, such as Yunnan and Guizhou, hold low values of monitoring data, mainly due to the low level of industrialization and high self-healing performance of the environment. Thus, the impact of atmospheric pollution incidents is treated differently by the AQI-based coefficient. 

**Figure 3:**
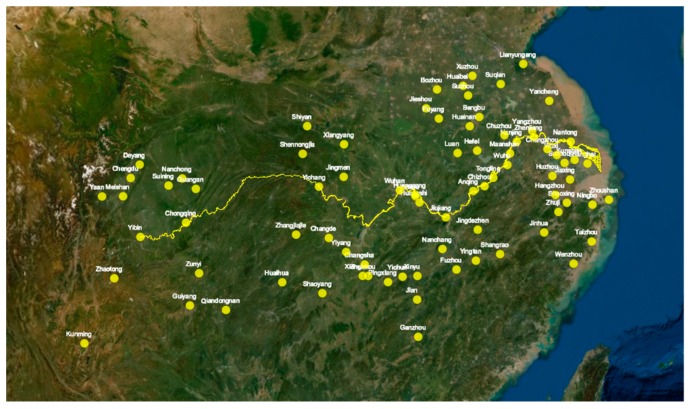
Distribution of cities mentioned in the news. Data source: Google Earth.

The spatial variation of incidents is marked in the YRB map along the Yangtze River (in Figure 3). There is an obvious agglomeration trend of the incidents along the lower reaches. The most densely distributed areas are the Yangtze River Delta, especially some coastal cities (Shanghai, Suqian, Jinhua, etc.), followed by the urban clusters in the middle reaches. The marked cities in the upper reaches are sparsely distributed. The synergistic pollution effect is also witnessed in the middle reaches of the Yangtze River. Relatively, the indicators of economic development and environmental condition are obviously showing the same trend. Overall, high air pollution is concentrated in the Zhejiang-Jiangsu-Anhui province Yangtze River Delta urban agglomeration, the Wuhan-Nanchang metropolitan area, the Changsha-Zhuzhou-Xiangtan urban agglomeration, and the Chengdu-Chongqing urban agglomeration. The low pollution incidents are located in the upstream cities (Yunnan, Guizhou) which are generally poorly developed. Meanwhile, the natural topography and environmental purification promote the transport and degradation of pollutants.

### 3.2. Frequencies Analysis

To some extent, high-frequency keywords show the general characteristics of atmospheric pollution. In this section, the high-frequency keywords and subjects of incidents are discussed. A total of 63 core words representing the main characteristics and human effects of air pollution incidents are selected to analyze the category and scope of the sample. Some low-frequency keywords are removed to make the traits of core words more distinct and evident. Some of the high-frequency keywords (found more than 8 times) are shown in Table 1. 

The keywords can be roughly divided into categories. “Enterprise pollution” (256), “coal-burning pollution” (97) and “traffic pollution” (34) show the main types of air pollution sources, which are followed by some typical pollutants such as “dust” (61), “waste gas” (46), “fugitive dust” (37) and “organic waste gas” (14). “Particulate matter” (13) gradually becomes a major concern of the public and the chief component of “air pollution sources” (21). “Sulfur dioxide” (16) and “nitrogen oxides” (12) are the most common chemical pollutants dispersed in the air and related to the pollution incidents. Human behaviors, the main factor and indirect cause of air pollution, are explicated by “incomplete environmental approvals” (65), “excessive discharge” (61), “direct discharge” (57), “open-air operation” (53), etc. Keywords also represent some relevant facilities such as “coal-fired equipment” (72) and “dust facility” (55). As traffic conditions are stressed to be the main causes of urban pollution, representative vehicles (e.g., “diesel vehicle”, “higher-emission vehicle”, “car”, “truck”, “bus”) have been mentioned frequently. Meanwhile, keywords with a lower frequency, such as “sprinkling operation” (8), refer to the high emission work in a factory, workshop, etc., mainly related to some representative operation processes. 

**Figure 4:**
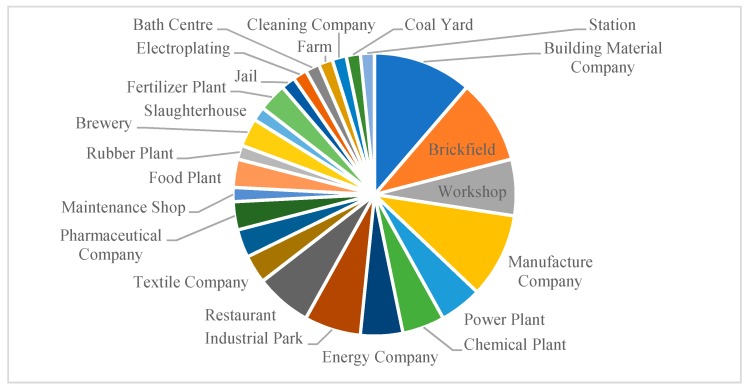
The distribution of pollution subjects.

The keywords of incident subjects are separated to analyze the regional characteristics of pollution triggers. In addition to “enterprise pollution” with the maximum frequency, manufacturing enterprises or factories contribute significantly to atmospheric pollutants. Figure 4 shows some lower-frequency keywords reflecting the types of enterprises, such as “building material company” (7), “brickfield” (6) and “manufacture company (6). A primary analysis to explore the distribution of pollution incidents inside the enterprises (workplaces) was visualized. The enterprises can be classified into 6 types according to their characteristics. Industrial enterprises (e.g., “building material company”, “brickfield”) are the main subjects of air pollution accounting for about 60.66% of the total subjects, while catering enterprises (e.g., “restaurant”, “workshop brewery”) constitute 24.59% of the incident subjects. Service enterprises (“maintenance shop”), agricultural enterprises (“slaughter house”), medical enterprises (“pharmaceutical company”) and transportation (“station”) also produce a number of pollutants. Notably, for some factories such as raw material factories, the contaminants are large and the requirements for environmental protection facilities are extremely strict. Other enterprises with a small scale of products, such as “workshop”, also suffered from problems, including insufficient coal quality, lack of prevention and control measures, and scattered pollution sources.

### 3.3. Correlation Matrix

A correlation matrix is a table showing the correlation coefficient between variables. Each cell in the table shows the correlation between two variables calculated by weighted Pearson’s correlation coefficient. The significance of various keywords is also shown in Table 2, where the significant correlation at 0.01 level is marked with "* *" and a significant correlation at 0.05 level is marked with "*". The main diagonal going from the top left to the bottom right represents that variables always perfectly correlate with themselves. This matrix is symmetrical, so only half of the two (significance and correlation) matrixes are reserved. The right upper part of the matrix represents the correlation coefficient and the lower left part indicates the significance. The whole table is too wide (63 by 63) and so only some of the high-coefficient keywords pairs are shown in Table 2. 

“Incomplete environmental approval” is highly correlated with several keywords. Among them, the highest correlation, with “dust”, is 0.904, which indicates that the control of dust pollution depends on effective supervision by governments [23]; otherwise, driven by economic interests, entrepreneurs may choose short-term economic income instead of paying for environmental costs. Then, “irregular operation” also holds a higher correlation coefficient with “incomplete environmental approval” (0.88). Obviously, the regulatory measures can reduce the possibility of illegal production and instruct workers to behave themselves. “Dust treatment facility” has a correlation with “incomplete environmental approval” (0.87), which indicates that the approval process puts forward requirements for relevant facilities, such as promoting dust-removal facilities and advancing the efficiency of dust processing. There are two main types of “excessive discharge”. One is active emissions, with “excessive discharge” and “direct discharge” having a correlation coefficient of more than 0.87. For large-scale production, the waste gas of the enterprise is directly discharged into the atmosphere without treatment. The other is passive, and the coefficient between “excessive discharge” and “dust treatment facility” is 0.813, which indicates that the damage of polluting facilities is the main cause of gas leakage. Due to the complexity of correlation analysis, further clustering of keywords is conducted.

### 3.4. Clustering

**Figure 5:**
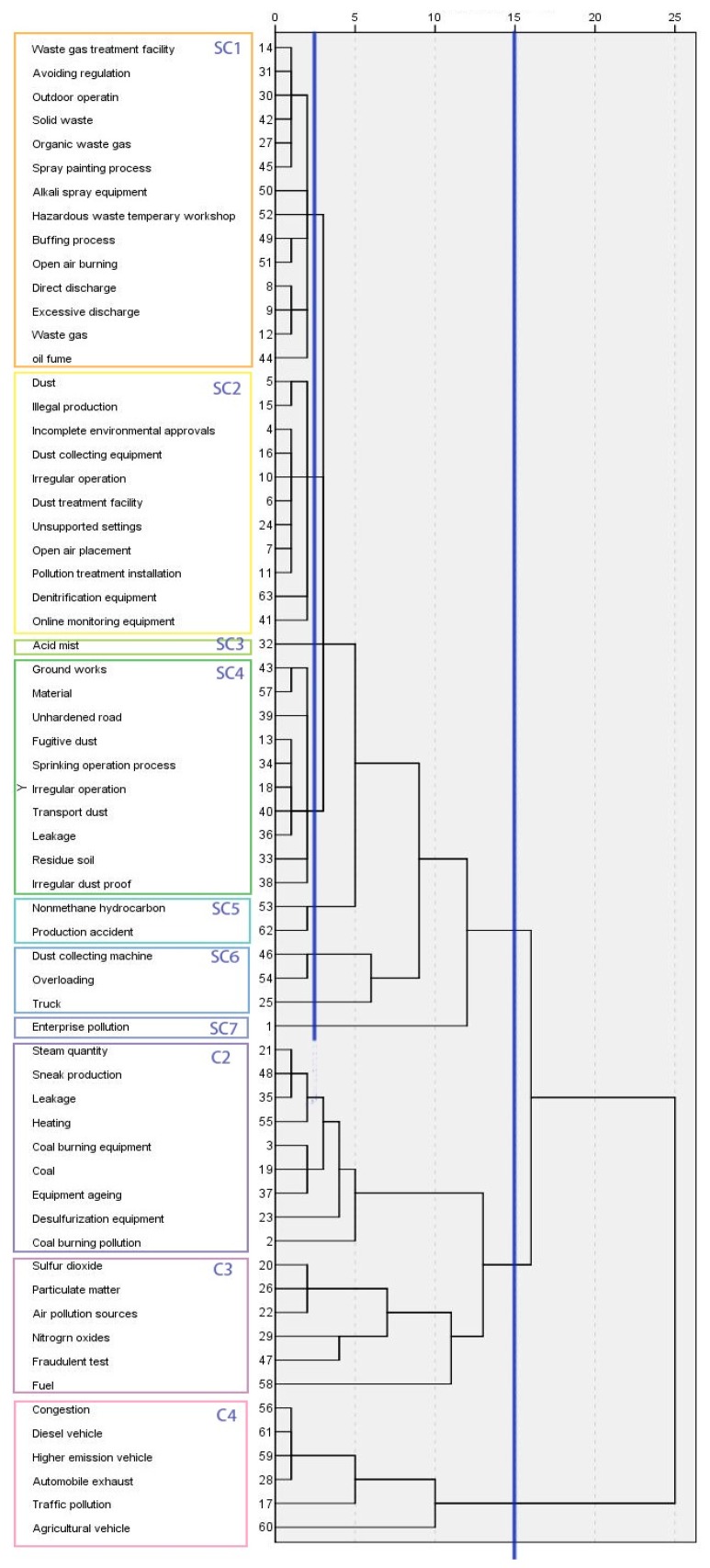
Dendrogram of keywords.

The 63 keywords obtained are divided into 4 clusters (as shown by the vertical line on the right side of Figure 4) according to hierarchical clustering techniques [48]. In each cluster, a keyword that represents the main theme is selected as a central word to explain the characteristics of that group. Clusters 1, 2, and 4 (C1, C2, C4) indicate the human effects and causes of “enterprise pollution”, “coal-burning pollution” and “traffic pollution”, while Cluster 3 (C3) lists some air-pollution sources resulting from environmental incidents, as shown in Figure 5.

- C1. Enterprise pollution and main pollutants

C1 is the largest group among all clusters. The complex and significant contents make it difficult to understand internal relations and characteristics. Thus, it generates more intersections and sub-clusters (SCs). The guides (blue line in Figure 5) are transferred to 2.5, and then six SCs are divided.

SC1 contains the most SCs in C1. “Waste gas” and “treatment facility” are the core keywords in this group indicating that “open-air burning” and “outdoor operation” (e.g., spraying painting process, buffing process) occur in many factories. Previous research [32] has focused more on the emission of industries, however the illegal (subjective and objective) behavior of some small workshops is also the main source of pollution gas emissions. According to the database, some small enterprises still have limitations on the understanding of the effect of illegal production, especially in some underdeveloped regions [15]. Environmental protection has always been a slogan rather than a personal act [9]. Before the generation of new energy, the use of fossil fuels is still the main energy supply way to provide the driving force of social development. Objective (direct emission) and subjective (excessive emissions) behaviors are the main causes of enterprise pollution. The former denotes some inevitable emissions, which can only be reduced by filtering devices to decrease the pollutants; the latter indicates the fluke mentality of the manager. In particular, the frequency of the “buffing process” is relatively high, so the prevention and control of pollutants should be strengthened in the production workshops. 

The core words of SC2 and SC3 are “dust” and “acid mist”. The generation devices of dust pollutants are mentioned (e.g., dust collecting equipment”, “denitrification equipment”, “online monitoring equipment”). Machine failure or shutdown often occurs due to subjective or objective reasons (“illegal production”, “illegal production”, “open-air placement”). The dust pollution in factories could be attributed to the placement and collection (or treatment) and, especially, uncovered preservation and direct discharge are important factors leading to dust pollution. 

SC4 focuses on the pollution caused by enterprise transportation (or on the road). General studies usually utilize road density (RD) to assess the impact of traffic on the environment [26]. Moreover, “fugitive dust”, “groundworks” and “unhardened road” also show irregular pollution characteristics. Pollutants accumulate and are produced gradually in the process of storage, transportation, outdoor work, etc. “Leakage” indicates the main method of generating dust unconsciously in possible transportation accidents. The influence of traffic congestion, traffic mode, and road capacity on the whole carbon footprint life cycles in the green supply chain has been discussed [49]. “Sprinkling operation process” is an effective way to solve dust problems. The average frequency of the keywords in SC4 is above 30, which indicates that the problems displayed by these kind of incidents can be easily ignored in production. 

SC5 and SC6 represent one relatively independent type of pollution incident: production accidents. “Nonmethane hydrocarbon” is mainly derived from the pollutants of anthropogenic emissions due to the high harmfulness. Meanwhile, high humidity and a certain amount of nitrides promote the formation of secondary organic aerosols [33]. Generated by sunlight under certain circumstances, photochemical smog causes serious negative impacts on the environment and human health. “Production accident” shows the serious consequences of irregular behavior with a high frequency of keywords: air pollution, direct threat to the enterprises, employee safety, etc. 

SC6 indicates illegal transportation during the process of manufacturing. “Truck” acts an extremely important role in the transportation of polluting raw materials in enterprises. However, illegal operations such as “overloading” and “unsealed preservation” make the process of dust collection extremely difficult. As the most important keyword, “enterprise pollution” forms SC7 independently.

- C2. Coal-burning and air pollutions

C2 includes the causes of “coal-burning pollution” from two main ways: coal-fired production and coal-fired heating. Zhou et al [10] utilized a geographical detector method to suggest that industrial soot emissions are primary contributors to air pollution. “Desulfurization equipment” and other similar purification equipment for emission gas in the coal-fired process are utilized abnormally. The efficiency of fuel and waste gas purification has been significantly reduced in some small factories. These entrepreneurs continue to ignore the policy for natural benefits and “sneak production” happens frequently. As to residential consumption, although there is no central heating policy for the provinces in the YRB, non-standardized coal-fired heating raises social issues.

- C3. Various air pollutions

C3 addresses the pollutions that have been monitored by relevant departments, such as “fine particular matter”, “sulfur dioxide”, “nitrogen oxides”, and other “air pollution sources”. The pollutants produced by “fuel” are addressed, especially for fossil fuels, which can produce carbon dioxide, sulfur dioxide, nitrogen dioxide, carbon monoxide, inhalable particulate matter, and other air-polluting substances. They can easily cause a greenhouse effect, acid rain, visibility reduction, and other meteorological disasters. Some researchers treat carbon as a footprint to follow the pollution path based on monitoring data such as the AOI or statistical reports. However, some hidden human effects are ignored. For instance, businesses and individuals try “fraudulent tests” to evade supervision, which is the behavior that lacks both common sense and social responsibility.

- C4. Traffic and air pollution

From the bottom to the top, C4 explores the causes of pollution from the perspective of transportation. Poor-visibility weather conditions and high mortality of newborns have a strong relationship with traffic emissions [50]. Current studies have stated that transportation is a major source of air pollution in urban areas, which are always divided into two parts including public transportation and individual traffic [51]. In line with previous studies, the high-frequency keywords “high emission vehicle” and “diesel vehicle” address the main traffic pollution source affecting the urban atmospheric environment. The use of fossil fuels and unqualified tail gas filtration may be the major causes. Meanwhile, with population centralization and urban integration, traffic “congestion” has also aggravated the trend of atmospheric pollution. Especially, “agricultural vehicles”, due to diesel fuel consumption and low fuel utilization, also cause emissions of exhaust gases (SO_2_, NO_X_, etc.)

### 3.5. Visualization Mapping

**Figure 6:**
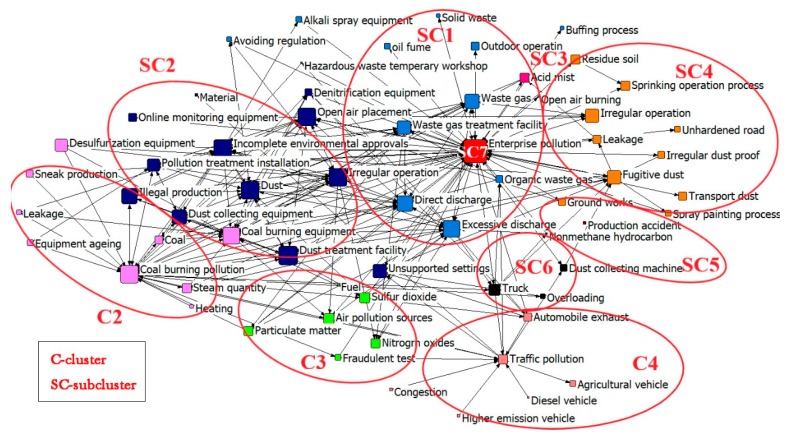
Visualization map based on MDS and node centrality.

To make further explorations, a dendrogram is utilized to visualize keyword interactions. The connections and strengthened links are presented by two-way arrows, while the degree of each node symbolizes a keyword’s occurrence time, as shown in Figure 6.

C1, divided into 7 SCs, contains approximately two-thirds of the keyword clustering network. This indicates that C1 reflects the main causes and human influences of air pollution in the YRB. Detailed types and characteristics of air pollution incidents are shown in 7 SCs. Meanwhile, C2 and C4 are closer to C1 in the visualization map. This indicates that they have greater correlations with C1, while C3 has relatively average relationships with the others.

SC7 (“enterprise pollution”) is the core word of the entire network diagram. It exhibits high values of frequency and centrality, and the node degree is the largest. Keywords that are directly related to SC7 are mainly in SC1. These keywords not only illustrate the high-frequency pollutants (waste gas, etc.) but also explain the causes of pollution (“direct discharge”, “excessive discharge”). This indicates that enterprise pollution is still an essential trigger to the deteriorating air quality in a certain area [52]. Direct or indirect factors such as improper supervision and pollutant discharge are the most important causes. It is worth noting that several types of pollutants (“solid waste”, “oil fume”, etc.) are at the top of the network map. Factors such as “outdoor operation” are only associated with enterprise pollution. 

Except for SC1, SC2 is in the immediate vicinity of the core word “enterprise pollution” and holds the highest internal correlation. In the entire network, the high-frequency keywords involved in SC2 hold the highest average centrality. “Dust pollution” relates to almost all the factors in SC2. The operation principle of equipment associated with dust is explained in this group. In particular, “open-air placement” and “irregular operation” are important mediators of SC1 and SC2. These two irregular operations often form synergistic effects on the generation of pollution, especially for the production of aerosols. 

A coal-burning pollution network is formed in C2, which is closely linked to SC2. This indicates that when businesses conduct coal burning to obtain energy, pollutants are mainly produced through indirect methods (harmful dust). Northern cities experience more dark winters than southern China, mainly due to the coal-fired heating and monsoon climate [11]. Terrible environmental impacts do not occur directly but are accompanied by a multiple-pollution phenomena. C2 is far from the central word and at the edge of the keyword network, illustrating that it holds the least correlation with enterprise pollution, but refers to the use of residents. Also, C2 forms a relatively independent pollution classification, which is mainly related to the characteristics of supply heating and coal burning in small workshops.

SC4 (with the central word “fugitive dust”) is directly linked to SC1. The frequency and centrality of the keywords in this cluster are relatively small. With a position on the edge of the network, the contribution of the causes mentioned in this group is relatively small. Compared with SC2, the dust mainly refers to a small amount of dirt or ash resulting from the irregular stacking (on open space) or illegal storage (on means of transport), which are rarely mentioned in previous studies. Some high-frequency keywords (“irregular operation”, etc.) are directly related to SC1, but few keywords are associated with other clusters. Moreover, the center of SC4 (“fugitive dust”) slightly deviates, as the correlation within the sub-cluster is quite different. For example, the main generating place and mode of fugitive dust have little to do with some enterprise processes (e.g., “sprinkling operation process”).

SC5 and SC6 are small in scale and cover low impact ranges. However, as intermediary factors, they link SC1, SC4, and C4. To a certain extent, the keywords in SC5 and SC6 are the causes of pollutions mentioned in SC1, SC4, and C4. SC5 and SC6 interact and form the combined action as well, mainly referring to the atmospheric pollutants (“nonmethane hydrocarbon”) produced by trucks. NOx emission from motor vehicles can greatly hinder the oxygen transport function of the human body. Exhaust emissions have a seriously harmful effect on pedestrians (especially traffic police who have a much lower average life than urban people [12]). C4 is based on traffic pollution and shows obvious divergent characteristics. Its key factors are mostly related to the core words, and there is no relationship with other SCs. This result reflects that the vehicles and traffic conditions within the clusters are direct and independent causes of traffic pollution.

C3 is a cluster with no obvious core words. It is mainly composed of various atmospheric pollution sources that share a relatively average centrality. The cluster is connected to other ones, especially for SC2 and SC1. The role of dust, NO_x,_ and SO_2_ in the formation of aerosols is further explained [53]. It illustrates that enterprise pollution and dust pollution share the maximum contribution to C3 (air pollutants). Unsupported settings and dust treatment facilities are closely related to the generation of atmospheric pollutants.

The high-frequency keywords reflect that “enterprise pollution” has the greatest impact on the air quality in the YRB. Also, the overwhelming majority of environmental problems in these enterprises (existing in each production process) are the human effects. The consequences are often serious, posing a great threat to the health of residents (e.g., cancerous village, pneumoconiosis). In addition, the lack of effective regulatory mechanisms (e.g., lack of education, publicity and insufficient efforts) is also the main cause. Moreover, the highly polluting vehicles still in use and the coal-based energy structure are sources of atmospheric pollution as well.

## 4. Conclusions

Building a healthy, green and energy-saving golden waterway is becoming the desire of residents in the YRB. A co-word analysis method is innovatively utilized to crawl through more than 3000 pieces of raw data and visualize the atmospheric pollution of the YRB. Statistical analysis of the keywords verifies that meteorological and natural factors are responsible for air pollution, and could also better uncover the spatial distribution of incidents. Furthermore, the interaction and clustering of factors shows that the generation of pollutants is correlated and often occurs in parallel. The harmful substances in the production process of businesses have become the most important environmental issue in the air pollution in the YRB. 

As to the subject consciousness of air pollution in the YRB, two main methods are reported in this research: illegal (active) and negligent (passive) emissions. Legislation and publicity can effectively avoid the occurrence of illegal emissions, and improve the legal awareness of incident subjects. Secondly, major types of pollution enterprises are found, including material enterprises, brick factories, and chemical plants. Irregular operation is also one of the most important sources of air pollution. The government must strengthen supervision, especially over small and medium-sized enterprises, to avoid illegal production, transportation, and emission processes. Thirdly, four groups are mentioned in the process of clustering. Besides some characteristics of traditional pollution types (enterprise pollution, coal-burning pollution, and traffic pollution), some emerging types of pollutions, such as PM_2.5_, are reported. Meanwhile, network characteristics of factors and clusters are also analyzed to expound the synergism of different incidents to air pollution.

This paper collects the incidents reported by mainstream media news and government reports, which makes a reasonable explanation for air pollution in the YRB. However, due to limitations of the number of samples, further research may concentrate on supplementation of the data set (e.g., self-media, local law). Meanwhile, an appeal should be made for more experts and scholars to conduct research into a pollution database collection of the YRB.

## Figures and Tables

**Table 1:** Categories of high-frequency keywords (found more than 8 times).

Keyword	Freq.	Keyword	Freq.	Keyword	Freq.
Enterprise pollution	256	Irregular operation	43	Automobile exhaust	17
Coal-burning pollution	97	Irregular operation	43	Sulfur dioxide	16
Coal-burning equipment	72	Pollution treatment installation	40	Organic waste gas	14
Incomplete environmental approvals	65	Waste gas treatment facility	38	Steam quantity	13
Dust	61	Fugitive dust	37	Truck	13
Excessive discharge	61	Traffic pollution	34	Particulate matter	13
Direct discharge	57	Illegal production	32	Unsupported settings	12
Dust treatment facility	55	Dust collecting equipment	30	Nitrogen oxides	12
Open air placement	53	Air pollution sources	21	Desulfurization equipment	11
Waste gas	46	Coal	18	Outdoor operation	8

**Table 2:** Part of the correlation matrix.

Keywords	Coal burning Equipment	Incomplete Environmental Approvals	Dust	Dust Treatment Facility	Open Air Placement	Direct Discharge	Excessive Discharge
Coal burning equipment	1.000	0.526 *	0.586 **	0.418 **	0.411 **	0.391 *	0.297 *
Incomplete environmental approvals	0.176	1.000	0.904 **	0.873 **	0.888 **	0.832 **	0.783 **
Dust	0.007	0.000	1.000	0.760 **	0.802 **	0.789 **	0.771 **
Dust treatment facility	0.009	0.000	0.760	1.000	0.845 **	0.847 **	0.813 **
Open air placement	0.008	0.001	0.000	0.000	1.000	0.766 **	0.746 **
Direct discharge	0.175	0.001	0.000	0.000	0.000	1.000	0.872 **
Excessive discharge	0.035	0.002	0.000	0.000	0.000	0.000	1.000

“*” means that the corresponding keywords are generally relevant. “**” means that the corresponding keywords are extremely relevant.

## References

[1] Hnatyshyn M. (2018). Decomposition analysis of the impact of economic growth on ammonia and nitrogen oxides emissions in the European Union. J. Int. Stud..

[2] Galnaitytė A., Kriščiukaitienė I., Baležentis T., Namiotko V. (2017). Evaluation of technological, economic and social indicators of different farming practices in Lithuania. Econ. Sociol..

[3] Kampa M., Castanas E. (2007). Human health effect of air polution-Enviro Polution. Environ. Pollut..

[4] Qin T., Yang P., Groves C., Chen F., Xie G., Zhan Z. (2018). Natural and anthropogenic factors affecting geochemistry of the Jialing and Yangtze Rivers in urban Chongqing, SW China. Appl. Geochemistry.

[5] Chen B., Yang S., Xu X.D., Zhang W. (2014). The impacts of urbanization on air quality over the Pearl River Delta in winter: Roles of urban land use and emission distribution. Theor. Appl. Climatol..

[6] Fan Q., Yang S., Liu S. (2019). Asymmetrically Spatial Effects of Urban Scale and Agglomeration on Haze Pollution in China. Int. J. Environ. Res. Public Health.

[7] Yu Y., Wang J., Yu J., Chen H., Liu M. (2019). Spatial and temporal distribution characteristics of PM 2.5 and PM 10 in the urban agglomeration of China’s Yangtze river delta, China. Polish J. Environ. Stud..

[8] Liu Y., Dong F. (2019). How Industrial Transfer Processes Impact on Haze Pollution in China: An Analysis from the Perspective of Spatial Effects. Int. J. Environ. Res. Public Health.

[9] Wu J., Zhang P., Yi H., Qin Z. (2016). What causes haze pollution? An empirical study of PM2.5 concentrations in Chinese cities. Sustainability.

[10] Zhou C., Chen J., Wang S. (2018). Examining the effects of socioeconomic development on fine particulate matter (PM2.5) in China’s cities using spatial regression and the geographical detector technique. Sci. Total Environ..

[11] Xu B., Luo L., Lin B. (2016). A dynamic analysis of air pollution emissions in China: Evidence from nonparametric additive regression models. Ecol. Indic..

[12] Tian X., Dai H., Geng Y., Wilson J., Wu R., Xie Y., Hao H. (2018). Economic impacts from PM2.5 pollution-related health effects in China’s road transport sector: A provincial-level analysis. Environ. Int..

[13] Peters H.P.F., van Raan A.F.J. (1993). Co-word-based science maps of chemical engineering. Part I: Representations by direct multidimensional scaling. Res. Policy.

[14] Wang T., Jiang F., Deng J., Shen Y., Fu Q., Wang Q., Fu Y., Xu J., Zhang D. (2012). Urban air quality and regional haze weather forecast for Yangtze River Delta region. Atmos. Environ..

[15] Liu H., Fang C., Zhang X., Wang Z., Bao C., Li F. (2017). The effect of natural and anthropogenic factors on haze pollution in Chinese cities: A spatial econometrics approach. J. Clean. Prod..

[16] Lu D., Xu J., Yang D., Zhao J. (2017). Spatio-temporal variation and influence factors of PM2.5concentrations in China from 1998 to 2014. Atmos. Pollut. Res..

[17] Li L., Qian J., Ou C.Q., Zhou Y.X., Guo C., Guo Y. (2014). Spatial and temporal analysis of Air Pollution Index and its timescale-dependent relationship with meteorological factors in Guangzhou, China, 2001-2011. Environ. Pollut..

[18] Bao J., Yang X., Zhao Z., Wang Z., Yu C., Li X. (2015). The Spatial-Temporal Characteristics of Air Pollution in China from 2001–2014. Int. J. Environ. Res. Public Health.

[19] Mukhtarova K.S., Trifilova A.A., Zhidebekkyzy A. (2016). Commercialization of green technologies: An exploratory literature review. J. Int. Stud..

[20] Jiang L., Zhou H.F., Bai L., Zhou P. (2018). Does foreign direct investment drive environmental degradation in China? An empirical study based on air quality index from a spatial perspective. J. Clean. Prod..

[21] Ma Y.R., Ji Q., Fan Y. (2016). Spatial linkage analysis of the impact of regional economic activities on PM2.5 pollution in China. J. Clean. Prod..

[22] Ma T., Duan F., He K., Qin Y., Tong D., Geng G., Liu X., Li H., Yang S., Ye S. (2019). Air pollution characteristics and their relationship with emissions and meteorology in the Yangtze River Delta region during 2014–2016. J. Environ. Sci..

[23] Wang W., Yu J., Cui Y., He J., Xue P., Cao W., Ying H., Gao W., Yan Y., Hu B. (2018). Characteristics of fine particulate matter and its sources in an industrialized coastal city, Ningbo, Yangtze River Delta, China. Atmos. Res..

[24] Mou Y., Song Y., Xu Q., He Q., Hu A. (2018). Influence of Urban-Growth Pattern on Air Quality in China: A Study of 338 Cities. Int. J. Environ. Res. Public Health.

[25] Koziuk V., Dluhopolskyi O., Farion A., Dluhopolska T. (2018). Crony sectors as a barrier to economic well-being and ecologization (Case of Ukraine). Econ. Sociol..

[26] He L., Liu Y., He P., Zhou H. (2019). Relationship between Air Pollution and Urban Forms: Evidence from Prefecture-Level Cities of the Yangtze River Basin. Int. J. Environ. Res. Public Health.

[27] Fan H., Zhao C., Yang Y. (2020). A comprehensive analysis of the spatio-temporal variation of urban air pollution in China during 2014–2018. Atmos. Environ..

[28] Song C., Wu L., Xie Y., He J., Chen X., Wang T., Lin Y., Jin T., Wang A., Liu Y. (2017). Air pollution in China: Status and spatiotemporal variations. Environ. Pollut..

[29] Zhan D., Kwan M.-P., Zhang W., Wang S., Yu J. (2017). Spatiotemporal Variations and Driving Factors of Air Pollution in China. Int. J. Environ. Res. Public Health.

[30] He D., Gao P., Sun Z., Lau Y.Y. (2017). Measuring water transport efficiency in the Yangtze River Economic Zone, China. Sustainability.

[31] Cheng S., Xie J., Xiao D., Zhang Y. (2019). Measuring the Environmental Efficiency and Technology Gap of PM2.5 in China’s Ten City Groups: An Empirical Analysis Using the EBM Meta-Frontier Model. Int. J. Environ. Res. Public Health.

[32] Zou B., You J., Lin Y., Duan X., Zhao X., Fang X., Campen M.J., Li S. (2019). Air pollution intervention and life-saving effect in China. Environ. Int..

[33] Liu C., Chen R., Zhao Y., Ma Z., Bi J., Liu Y., Meng X., Wang Y., Chen X., Li W. (2017). Associations between ambient fine particulate air pollution and hypertension: A nationwide cross-sectional study in China. Sci. Total Environ..

[34] Ding Y., Chowdhury G.G., Foo S. (2001). Bibliometric cartography of information retrieval research by using co-word analysis. Inf. Process. Manag..

[35] Ravikumar S., Agrahari A., Singh S.N. (2015). Mapping the intellectual structure of scientometrics: A co-word analysis of the journal scientometrics (2005–2010). Scientometrics.

[36] Benckendorff P., Zehrer A. (2013). A network analysis of tourism research. Ann. Tour. Res..

[37] Leung X.Y., Sun J., Bai B. (2017). Bibliometrics of social media research: A co-citation and co-word analysis. Int. J. Hosp. Manag..

[38] Vogel R., Güttel W.H. (2013). The dynamic capability view in strategic management: A bibliometric review. Int. J. Manag. Rev..

[39] He Q. (1999). Knowledge Discovery Through Co-Word Analysis. Libr. Trends.

[40] Wu D., Song Y., Xie K., Zhang B. (2018). Traits and causes of environmental loss-related chemical accidents in China based on co-word analysis. Environ. Sci. Pollut. Res..

[41] Jiang Z., Tao Y. (2018). Co-word analysis and bibliometric visualization of translation quality literature: research topics and trends in the Chinese mainland (1997–2016). Asia Pacific Transl. Intercult. Stud..

[42] Armah F.A., Yawson D.O., Pappoe A.A.N.M. (2010). A systems dynamics approach to explore traffic congestion and air pollution link in the city of Accra, Ghana. Sustainability.

[43] Tao Z., Kokas A., Zhang R., Cohan D.S., Wallach D. (2016). Inferring atmospheric particulate matter concentrations from Chinese social media data. PLoS One.

[44] Jiang W., Wang Y., Tsou M.H., Fu X. (2015). Using social media to detect outdoor air pollution and monitor air quality index (AQI): A geo-targeted spatiotemporal analysis framework with sina weibo (Chinese twitter). PLoS One.

[45] Lee P.-C., Su H.-N. (2010). Investigating the structure of regional innovation system research through keyword co-occurrence and social network analysis. Innovation.

[46] Aria M., Cuccurullo C. (2017). bibliometrix: An R-tool for comprehensive science mapping analysis. J. Informetr..

[47] Che H., Zhang X., Li Y., Zhou Z., Qu J.J., Hao X. (2009). Haze trends over the capital cities of 31 provinces in China, 1981-2005. Theor. Appl. Climatol..

[48] Murtagh F., Legendre P. (2014). Ward’s Hierarchical Agglomerative Clustering Method: Which Algorithms Implement Ward’s Criterion?. J. Classif..

[49] Simionescu M., Bilan Y., Gedek S., Streimikiene D. (2019). The effects of greenhouse gas emissions on cereal production in the European Union. Sustainability.

[50] Lelieveld J., Evans J.S., Fnais M., Giannadaki D., Pozzer A. (2015). The contribution of outdoor air pollution sources to premature mortality on a global scale. Nature.

[51] Vafa-arani H., Jahani S., Dashti H., Heydari J., Moazen S. (2014). A system dynamics modeling for urban air pollution: A case study of Tehran, Iran. Transp. Res. PART D.

[52] Tang D., Zhang Y., Bethel B.J. (2019). An analysis of disparities and driving factors of carbon emissions in the Yangtze River Economic Belt. Sustainability.

[53] Sabetghadam S., Ahmadi-Givi F. (2014). Relationship of extinction coefficient, air pollution, and meteorological parameters in an urban area during 2007 to 2009. Environ. Sci. Pollut. Res..

